# Supplementary material for: Chemical potential of quasi-equilibrium magnon gas driven by pure spin current
Source: Nat Commun. 2017 Nov 17;8:1579. doi: 10.1038/s41467-017-01937-y (PMC5691177; doi:10.1038/s41467-017-01937-y)
Supplement: Supplementary file 1 — Supplementary Information [file 41467_2017_1937_MOESM1_ESM.pdf]

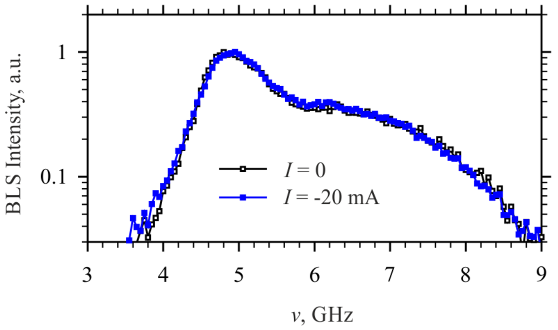

Supplementary Figure 1. Comparison of the shape of the spectra obtained at different currents. BLS spectra recorded at I=0 (open squares) and I=-20 mA, (solid squares). The spectrum obtained at I=0 is shifted in frequency by 50 MHz to compensate for the shift caused by the reduction of the effective magnetization and the Oersted field of the current. The BLS intensity obtained at I=-20 mA is multiplied by 1.32, to facilitate direct comparison. Note the absence of current-induced variations in the spectrum shape.

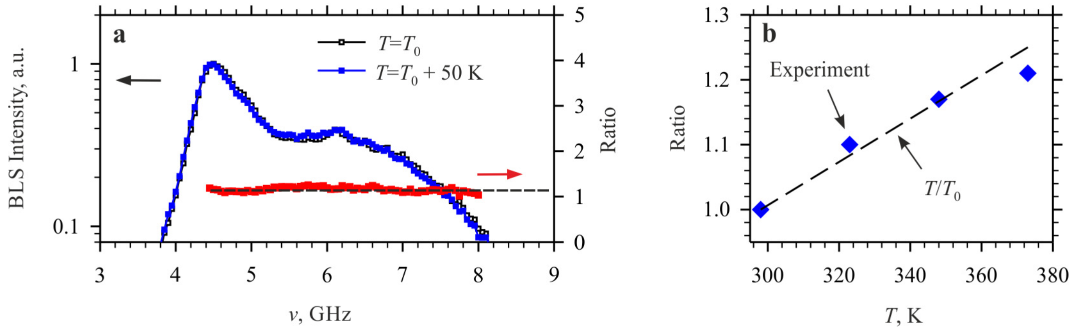

Supplementary Figure 2. Dependence of the BLS spectra on the temperature. BLS spectra obtained under the conditions of the uniform direct heating of the sample. **a,** BLS spectra recorded at T=T_0 and T=T_0+50 K, as labelled. The spectrum obtained at T=T_0 is shifted in frequency by 50 MHz to compensate for the shift caused by the reduction of the static magnetization due to the heating. The spectrum obtained at T=T_0+50 K is divided by 1.17 to facilitate direct comparison. Note the absence of variations in the spectrum shape. Solid red squares show the frequency-independent ratio between the two BLS spectra. Dashed line marks the mean value of the ratio, equal to 1.17. **b,** Temperature dependence of the ratio of the BLS spectra. Dashed line is T/T_0.

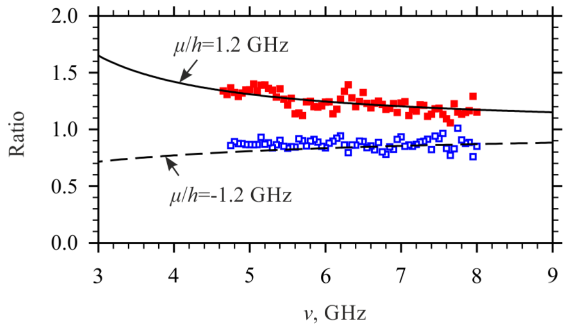

Supplementary Figure 3. Ratio of the BLS spectra obtained with and without current. Solid squares – I=5 mA, open squares – I=-5 mA. Solid curve shows the result of data fitting for I=5 mA by Eq. (1) with $T=T_0$ and μ/h=1.2 GHz. Dashed curve shows the ratio expected from the extrapolation of the linear dependence of the chemical potential at I>0 to I=-5 mA. Note that this curve noticeably deviated from the data at small frequencies, indicating that the effective chemical potential quickly saturates at I<0.
